# Parental eating attitudes and adolescent eating disorder severity: preliminary findings on orthorexic tendencies

**DOI:** 10.1007/s40519-026-01830-y

**Published:** 2026-02-27

**Authors:** Francesca Camardella, Valentina Pellegatta, Eleonora Poggiogalle, Maria Pia Casini, Federica Gigliotti, Samira Terlizzi, Angela Favaro, Patrizia Todisco, Paolo Meneguzzo, Lorenzo M. Donini

**Affiliations:** 1 Experimental Medicine Department, Food Science and Human Nutrition Research Unit, Sapienza University of Rome, Rome, Italy; 2 Department of Human Neuroscience, Unit of Child and Adolescent Neuropsychiatry, Sapienza University of Rome, Via Dei Sabelli 108, 00185 Rome, Italy; 3 Eating Disorder Unit, Casa di Cura “Villa Margherita” – KOS Group, Arcugnano, Vicenza, Italy; 4 Department of Neuroscience, University of Padova, Via Giustiniani 2, 35128 Padua, Italy; 5 Padova Neuroscience Center, University of Padova, Padua, Italy; 6 Psycho-Nutritional Center, Verona, Italy

**Keywords:** Eating Disorders, Adolescents, Orthorexia, Parental Influence, Nutritional Knowledge, Family Environment

## Abstract

**Introduction:**

Eating disorders (EDs) often emerge in adolescence and are influenced by family factors. Parental eating attitudes, including orthorexic tendencies, may be associated with adolescent ED severity. We examined parental orthorexic tendencies, cognitive rigidity, and nutritional knowledge in relation to adolescents’ ED symptoms.

**Methods:**

The sample comprised 59 adolescents (93.2% female; mean age = 18.34 ± 3.78 years) and their parents (36 dyads; 23 single parents). Parents completed measures of orthorexia (ORTO-R), cognitive rigidity (D-FLEX), and an ad hoc nutritional knowledge questionnaire; adolescents completed the EDE-Q. Analyses were conducted in the full sample and repeated in anorexia nervosa (AN).

**Results:**

In the full sample, higher maternal ORTO-R scores were associated with greater EDE-Q restraint (β = 0.29, p = 0.042) and shape concern (β = 0.31, p = 0.030). In fathers, older age was associated with adolescents’ shape (β = 0.32, p = 0.037) and weight concerns (β = 0.36, p = 0.018), whereas paternal ORTO-R was not significant. Mothers showed higher nutritional knowledge than fathers (U = 821.0, p = 0.013). No parent–child concordance emerged for BMI or eating psychopathology. Associations were no longer significant in AN-only analyses.

**Conclusion:**

Parental orthorexic tendencies showed small associations with adolescent ED severity in the full sample, with limited robustness in AN. Findings are preliminary and warrant replication in larger diagnostically stratified cohorts.

**Level of evidence:**

Level III, observational analytic study; cross-sectional design.

## Introduction

Eating disorders (EDs), including anorexia nervosa (AN), bulimia nervosa (BN), and binge eating disorder (BED), are severe psychiatric conditions characterised by disturbed eating behaviours and body image concerns, typically emerging during adolescence and disproportionately affecting young women [1]. EDs are associated with substantial psychological and physical morbidity and marked impairment in psychosocial functioning [2].

Familial factors play a central role in ED vulnerability and course, through a combination of genetic liability and environmental mechanisms such as modelling, reinforcement, and the transmission of food- and body-related beliefs [3, 4]. Parental eating behaviours and weight-related attitudes have been associated with adolescents’ disordered eating symptoms, and the family food environment may be particularly influential during adolescence, when autonomy, identity development, and emotion regulation are still consolidating. Despite this evidence, most studies have focused on maternal correlates, while paternal contributions remain comparatively understudied.

In recent years, increasing attention has been paid to orthorexic tendencies, conceptualised as a rigid and health-focused approach to eating that may overlap with restrictive dietary control and perfectionistic rule-based patterns [5, 6]. From an intergenerational perspective, parental orthorexic tendencies could shape family norms around “healthy” eating, potentially influencing adolescents’ restraint and shape/weight concerns, particularly in the context of existing ED vulnerability. Alongside orthorexic tendencies, cognitive factors such as cognitive rigidity and nutritional knowledge may contribute to the food-related climate within the family; however, evidence on these parental characteristics in clinical adolescent ED samples remains limited.

Therefore, this preliminary study aimed to (i) characterise orthorexic tendencies, cognitive rigidity, and nutritional knowledge in mothers and fathers of adolescents with EDs, (ii) examine associations between these parental variables and adolescents’ ED severity, and (iii) explore parent–child concordance in BMI and eating psychopathology. We hypothesised that higher parental orthorexic tendencies would be associated with greater adolescent ED severity, with stronger effects for mothers than for fathers. We also expected mothers to report higher nutritional knowledge than fathers, and anticipated limited parent–child concordance in BMI and ED psychopathology.

## Methods

### Participants

This cross-sectional study included 59 adolescents and young adults with a DSM-5 eating disorder and 95 parents recruited from three Italian clinical centres: Policlinico Umberto I (Rome; Unit of Research in Food Science), Casa di Cura Villa Margherita (Arcugnano, Vicenza), and the Regional Center for Eating Disorders at the University Hospital of Padova. Recruitment occurred over approximately 1 year starting in October 2022. Consecutive eligible patients evaluated in the participating services were invited to participate together with one or both parents. Participation was voluntary and written informed consent was obtained from all participants; for minors, parental consent and patient assent were obtained.

Eating disorder diagnoses were established by experienced clinicians using DSM-5 criteria based on a semi-structured diagnostic interview and routine clinical assessment. Eligible diagnoses included AN, BN, BED, and other specified feeding or eating disorders (OSFED). Inclusion criteria were age 15–30 years and ability to provide informed consent. Exclusion criteria were inability to provide written informed consent and/or to allow anonymous data processing.

For patients, demographic and clinical information (age, sex, ED diagnosis, illness duration, psychiatric/medical comorbidities, pharmacological treatment, and relevant clinical history) was obtained from medical records and clinical interviews. For parents, demographic variables and questionnaire responses were collected.The study protocol was approved by the Ethics Committee of Sapienza University (6522/22) and conducted in accordance with the Helsinki Declaration.

### Materials

Adolescents and young adults completed the Eating Disorder Examination Questionnaire (EDE-Q) [7], a self-report measure of ED psychopathology over the previous 28 days. Items are rated on a 7-point scale (0–6), yielding a global score and subscales (higher scores indicate greater severity).

Parents completed a structured questionnaire administered in person or remotely. Screening for eating disorder symptoms in parents was conducted using the SCOFF questionnaire [8], with two additional items included to screen for bulimic symptoms. Orthorexic tendencies were assessed using the ORTO-R [9], a 6-item self-report measure rated on a 4-point scale. Cognitive rigidity and attention to detail were evaluated with the Detail and Flexibility Questionnaire (D-FLEX) [10], a 24-item self-report measure rated on a 6-point scale (higher scores indicate greater cognitive inflexibility/attention to detail).

Parental nutritional knowledge (NK) was assessed using an ad hoc questionnaire comprising 29 multiple-choice items covering key domains of general nutrition literacy. Items assessed knowledge of dietary recommendations (e.g., intake frequency and portions of major food groups), nutrient composition of common foods (e.g., sugars, fats, salt, fibre, proteins), quality of fats and glycaemic properties, applied food choices in everyday contexts (e.g., restaurant meals, snacks, cooking methods), and common nutrition-related beliefs and misconceptions (e.g., “light” products, gluten-free foods, fibre and weight control). The questionnaire was developed based on Italian National Dietary Guidelines and World Health Organization recommendations and reviewed by two independent experts (nutrition and clinical psychology) to ensure content relevance and clarity. As the instrument was designed to assess factual knowledge rather than a latent psychological construct, no internal consistency indices were calculated. NK performance was treated descriptively and categorised into four ordinal levels (none correct; < 50% correct; > 50% correct; all correct) to facilitate interpretation. Given the absence of formal psychometric validation, NK findings were considered exploratory.

### Statistical plan

Analyses were cross-sectional and two-tailed with α = 0.05. Continuous variables are reported as mean ± SD and categorical variables as n (%). Comparisons between mothers and fathers were conducted using paired-sample tests within complete parental dyads (paired t-tests or Wilcoxon signed-rank tests, as appropriate). When only one parent was available, mothers and fathers were analysed descriptively.

Associations between parental variables and adolescents’ EDE-Q global and subscale scores were examined using Pearson’s correlations (mothers and fathers analysed separately). Variables showing bivariate associations at p < 0.10 were entered into linear regression models to evaluate independent associations with adolescent EDE-Q outcomes. Standardized regression coefficients (β) are reported as indicators of effect magnitude; no additional effect size indices and no correction for multiple comparisons was applied, given the exploratory nature and limited sample size. Analyses were performed using SPSS v25 (IBM, Armonk, NY).

Sensitivity analysis. Key regression models were repeated restricting the sample to adolescents with AN to evaluate robustness within the most homogeneous diagnostic subgroup.

## Results

The sample consisted of 59 adolescents and young adults with EDs (mean age: 18.34 ± 3.78 years; illness duration: 3.10 ± 3.01 years) and 95 parents (fathers: mean age 53.83 ± 5.22 years; mothers: mean age 51.45 ± 5.09 years). The patient group included 55 females (93.2%) and 4 males. Both parents participated for 36 patients (61%), whereas one parent was missing for 23 patients (13 fathers and 10 mothers). Diagnoses included AN (n = 41), BN (n = 7), BED (n = 6), and OSFED (n = 5). No significant differences were observed between patients with complete parental dyads and those with only one participating parent in terms of age, illness duration, or sex distribution; similarly, parental age, BMI, and education did not differ between these groups. Therefore, the full sample was analysed together.

The median NK category for both mothers and fathers corresponded to “more than half correct.” However, in within-family comparisons among complete dyads, mothers showed higher NK performance than fathers (p = 0.013), with a higher proportion of mothers in the highest category (60%) compared to fathers (35%).

Regarding SCOFF screening, 9 fathers (19.6%) and 14 mothers (28.6%) screened positive, with no significant difference between parents. Parental psychological characteristics are summarised in Table 1.

**Table 1:** Parental characteristics (mothers vs fathers)

Variable	Mothers (Mean ± SD)	Fathers (Mean ± SD)	Test (paired)	p
Age (years)	51.58 ± 5.68	54.08 ± 5.56	t = -3.08	0.004
BMI (kg/m^2^)	22.18 ± 3.15	23.97 ± 4.66	t = -2.13	0.041
ORTO-R total	2.66 ± 0.64	2.67 ± 0.44	t = -0.08	0.939
D-Flex Attention to Detail (OA)	30.25 ± 9.52	24.75 ± 8.07	t = 3.06	0.005
D-Flex Cognitive Rigidity (CI)	34.14 ± 8.29	31.72 ± 8.32	t = 1.16	0.252
D-Flex Total score (TS)	64.39 ± 15.79	56.47 ± 14.28	t = 2.22	0.033
Nutritional knowledge (NK)*	Median = 3	Median = 3	W = 16.0	0.011
SCOFF positive, n (%)	11 (30.6%)	6 (16.7%)	χ^2^ = 1.23	0.267

^*^NK is coded as 4 ordinal categories (1 = none correct; 2 =  < 50% correct; 3 =  > 50% correct; 4 = all correct)

### Parent–child concordance

Bivariate correlations were conducted to identify parental variables associated with adolescents’ EDE-Q outcomes. In mothers, ORTO-R scores were associated with adolescents’ restraint (r = 0.26, p = 0.046) and shape concern (r = 0.31, p = 0.015), with a trend for the global EDE-Q score (r = 0.25, p = 0.053). In fathers, age was associated with adolescents’ shape concern (r = 0.30, p = 0.017) and weight concern (r = 0.33, p = 0.007).

In regression analyses, higher maternal ORTO-R scores were associated with greater restraint (β = 0.29, p = 0.042) and shape concern (β = 0.31, p = 0.030), with a trend for weight concern (β = 0.24, p = 0.099). In fathers, older age was associated with shape concern (β = 0.32, p = 0.037) and weight concern (β = 0.36, p = 0.018), whereas paternal ORTO-R was not significantly associated with these outcomes (ps ≥ 0.77).

Results were not significant in AN-only models.

## Discussion

This preliminary study examined whether parental orthorexic tendencies, cognitive rigidity, and nutritional knowledge were associated with adolescents’ eating-disorder severity in a clinical sample. In the full sample, higher maternal orthorexic tendencies showed small associations with adolescents’ restraint and shape-related concerns, whereas paternal orthorexic tendencies were not associated with outcomes. In fathers, older age emerged as the most consistent correlate of adolescents’ shape/weight concerns. No meaningful parent–child concordance was observed for BMI or eating psychopathology. Paternal age should be interpreted cautiously and may reflect cohort-related differences in parenting styles, generational attitudes toward body image, or family roles, rather than a direct etiological factor.

The maternal findings are consistent with models highlighting the role of the family food environment and parental modelling in shaping eating-related beliefs and rules, particularly during adolescence [3, 4]. Notably, effect sizes were modest, suggesting that orthorexic tendencies may represent one correlate among multiple interacting factors rather than a primary driver of symptom severity. The absence of concordance in BMI and screening-based parental ED symptoms argues against a simple “symptom mirroring” interpretation and supports indirect pathways (e.g., normative family rules around “healthy eating,” reinforcement of dietary control).

Mothers displayed higher nutritional knowledge than fathers, which may reflect gendered caregiving roles and greater involvement in food-related responsibilities. However, nutritional knowledge was not correlated with orthorexic tendencies, supporting the view that orthorexia may reflect rigid rule-based beliefs more than evidence-based nutritional competence [6]. Sensitivity analyses restricted to anorexia nervosa did not replicate the full-sample associations, underscoring the need for larger diagnostically stratified studies to test diagnosis-specific patterns and account for unmeasured confounders.

Limitations include the cross-sectional design, modest sample size, heterogeneity of diagnoses, and the use of an ad hoc nutritional knowledge measure without formal psychometric validation. Replication in larger longitudinal cohorts is needed to clarify temporal direction and mechanisms.

## Conclusion

In this preliminary clinical sample, maternal orthorexic tendencies were modestly associated with adolescents’ eating-disorder severity, whereas paternal orthorexic tendencies were not, and paternal age emerged as a potential correlate of shape/weight concerns. The absence of parent–child concordance suggests that parental influence may operate through indirect mechanisms rather than direct symptom transmission. These findings require replication in larger, diagnostically stratified longitudinal studies.

## What is already known on this subject?

Previous research has shown that parental attitudes toward food, weight, and dietary control are associated with adolescents’ disordered eating, with maternal factors receiving more attention than paternal ones. Orthorexic tendencies have been conceptualized as a form of health-focused dietary rigidity that may overlap with restrictive eating patterns, yet their potential intergenerational relevance in clinical ED samples remains insufficiently explored.

## What this study adds?

In this preliminary clinical sample, higher maternal orthorexic tendencies showed small associations with adolescents’ restraint and shape-related concerns. In fathers, older age—rather than orthorexic tendencies—was associated with adolescents’ shape/weight concerns. These associations were attenuated in AN-only sensitivity analyses, highlighting the need for replication in larger, diagnostically stratified samples and suggesting that parental dietary rigidity may not map uniformly onto ED severity across diagnoses.

## Data Availability

The data that support the findings of this study are available from the corresponding author, upon reasonable request.
